# Comparison of the Microsatellite Distribution Patterns in the Genomes of Euarchontoglires at the Taxonomic Level

**DOI:** 10.3389/fgene.2021.622724

**Published:** 2021-02-26

**Authors:** Xuhao Song, Tingbang Yang, Xinyi Zhang, Ying Yuan, Xianghui Yan, Yi Wei, Jun Zhang, Caiquan Zhou

**Affiliations:** ^1^Key Laboratory of Southwest China Wildlife Resources Conservation (Ministry of Education), China West Normal University, Nanchong, China; ^2^Institute of Ecology, China West Normal University, Nanchong, China

**Keywords:** Euarchontoglires, genome, microsatellite distribution pattern, taxonomic features, functional annotation

## Abstract

Microsatellite or simple sequence repeat (SSR) instability within genes can induce genetic variation. The SSR signatures remain largely unknown in different clades within Euarchontoglires, one of the most successful mammalian radiations. Here, we conducted a genome-wide characterization of microsatellite distribution patterns at different taxonomic levels in 153 Euarchontoglires genomes. Our results showed that the abundance and density of the SSRs were significantly positively correlated with primate genome size, but no significant relationship with the genome size of rodents was found. Furthermore, a higher level of complexity for perfect SSR (P-SSR) attributes was observed in rodents than in primates. The most frequent type of P-SSR was the mononucleotide P-SSR in the genomes of primates, tree shrews, and colugos, while mononucleotide or dinucleotide motif types were dominant in the genomes of rodents and lagomorphs. Furthermore, (A)n was the most abundant motif in primate genomes, but (A)n, (AC)n, or (AG)n was the most abundant motif in rodent genomes which even varied within the same genus. The GC content and the repeat copy numbers of P-SSRs varied in different species when compared at different taxonomic levels, reflecting underlying differences in SSR mutation processes. Notably, the CDSs containing P-SSRs were categorized by functions and pathways using Gene Ontology and Kyoto Encyclopedia of Genes and Genomes annotations, highlighting their roles in transcription regulation. Generally, this work will aid future studies of the functional roles of the taxonomic features of microsatellites during the evolution of mammals in Euarchontoglires.

## Introduction

Microsatellites, or simple sequence repeats (SSRs) are tandem repetitions of relatively short DNA motifs present in perfect (P-SSR), compound (C-SSR), and imperfect (I-SSR) forms in nearly all known genomes (Du et al., 2018; Du et al., 2020). Polymorphic microsatellites have been widely utilized as popular molecular markers for studying neutral genetic variation in diverse fields, including individual identification (Huang et al., 2015), population genetics (Zepeda et al., 2019), and other genetic studies (Highnam et al., 2012; Aristizábal et al., 2018). Recently, SSR instability in functional genes has been shown to be associated with many human diseases, such as neurological disorders (Rohilla and Gagnon, 2017) and colorectal cancers (Yamamoto and Imai, 2019). In particular, SSRs could also play an important role in generating the genetic variation underlying the adaptive evolution of organisms. There are substantial data indicating that SSR mutability can affect gene regulation as well as transcription and protein function, which ultimately confer phenotypic flexibility/plasticity (Holder et al., 2015; Bagshaw et al., 2017; Press et al., 2018).

Aside from the ubiquity and functional significance of SSRs, tremendous progress has been made in characterizing the distribution patterns of SSRs in diverse eukaryotic genomes (Qin et al., 2015; Ding et al., 2017; Srivastava et al., 2019). Indeed, comparative genomics approaches have aided the exploration of microsatellite conservation footprints in eukaryotic species evolution. More specifically, previous analyses of SSRs within 136 insect genomes revealed that common genomic features of SSRs were detectable at the family level (Ding et al., 2017). Furthermore, an investigation of P-SSRs in 719 eukaryotic species revealed several taxon-specific P-SSR characteristics as well as some evolutionary differences in the context of length and GC content of these P-SSRs (Srivastava et al., 2019). Meanwhile, profound interspecific variability in SSR distribution patterns in genomes has also been reported in insects, which suggests that variation might play an important role in the adaptation and evolution of insects (Behura and Severson, 2012; Song et al., 2020). Microsatellites present various degrees of taxon-specific enrichment in different lineages; thus, comparative analyses of SSRs at different taxonomic levels could therefore provide insight into the significance of evolutionarily relevant SSRs.

Euarchontoglires is a superclade of placental mammals that includes primates, rodents, lagomorphs, tree shrews, and colugos. Dynamic evolutionary signatures of microsatellites in Euarchontoglires genomes may be present because many species in this group are characterized by their successful adaptive radiation to various ecological niches. Although over 150 Euarchontoglires genomes are now available in the GenBank database, the SSR distribution patterns have only been studied in a handful of species in this clade (Liu et al., 2017; Xu et al., 2018; Srivastava et al., 2019). Thus, a genome-wide characterization of the microsatellite distribution patterns in Euarchontoglires genomes remains to be completed. Here, we investigated the distribution patterns of SSRs in 153 species, representing five taxonomic orders of Euarchontoglires (Rodentia, Lagomorpha, Primates, Scandentia, and Dermoptera). Comparisons of the distribution patterns of SSRs among different taxonomic levels were made to characterize taxonomic patterns of the microsatellite distributions in Euarchontoglires. Furthermore, the potential functions of microsatellite-containing CDSs were further surveyed using Gene Ontology (GO) enrichment analysis and Kyoto Encyclopedia of Genes and Genomes (KEGG) pathway analysis. We present a detailed characterization of the taxon-specific distribution pattern of SSRs among 153 species and provides insight into the biological significance of SSRs in this clade.

## Materials and Methods

### Genome Dataset

Currently, the genomes of 153 species (57 primates, 88 rodents, four lagomorphs, three tree shrews, and one colugo) within Euarchontoglires are publicly available (Supplementary Table 1). All currently available Euarchontoglires genomes were downloaded from GenBank^1^ for microsatellite identification and analysis. Ambiguous nucleotides were removed from the genomes prior to analysis. Detailed taxonomic information of these organisms was gathered from the National Center of Biotechnology Information (NCBI) database. The features of these genomes are presented in Supplementary Table 1. Among the 153 genomes, 57 genomes (26 primates, 27 rodents, two lagomorphs, one tree shrew, and one colugo) were annotated with protein-coding genes accompanied by gff3 annotation files containing positional information for exons and introns (Supplementary Table 1). The hierarchical classification provided by TimeTree (Kumar et al., 2017) was downloaded as a Newick file and was used for visualization through the iTOL (interactive Tree of Life) web server (Letunic and Bork, 2016).

### Microsatellite Identification

According to the methods described previously (Qi et al., 2020), SSRs (i.e., P-SSRs, C-SSRs, and I-SSRs) were screened and localized using Krait v1.0.3 software (Du et al., 2018). Furthermore, the P-SSRs located within intergenic regions were further identified by a Python script according to the annotation file. Repeats with unit patterns of circular permutations and/or reverse complementation to each other were grouped together as a single type for statistical analysis as described previously (Xu et al., 2016). Overall, 5356 possible permutations of SSR motifs 1–6 bp in length were divided into 501 stand motif types as described by Srivastava et al. (2019). In addition, the relative positions of exons, introns, and CDSs were extracted from the annotation files for genomic annotation of P-SSRs, C-SSRs, and I-SSRs by Krait v1.0.3.

### Microsatellite Attribute Investigation

In this study, the prevalence of SSRs in the genome was assessed by SSR abundance (loci/Mb) and SSR density (bp/Mb) as described by Qi et al. (2016). The abundance of the 501 stand repeat motif types in each genome was calculated by a custom Python script. A heatmap was generated based on the density of all SSR motif classes in each organism as described by Srivastava et al. (2019) with slight modifications, which could reveal repeat class-specific enrichment trends among different taxon groups. First, we ranked all of the repeat motif classes based on their density in each species. Furthermore, we first gave −2 to those repeats that had a frequency of < 10 in a given organism, to reduce sampling bias. Next, we assigned scores of 3, 2, and 1 to repeats with the top 5, 20, and 35 ranks in the genome, respectively. Repeats in the bottom 20 ranks and with a frequency of at least 10 were given a score of −1. All other repeats were assigned a score of 0. A matrix was built using the score information, where each row represents an organism and the columns represent the repeated classes. The clustered matrix was visualized using TBtools (Chen et al., 2020). The color scale on the heatmap ranged from a maximum score of 3 (red) to a minimum score of −2 (blue).

The GC content of mono- to hexanucleotide P-SSRs was also calculated for the GC composition analysis in each organism using in-house Python scripts. Meanwhile, the preference analysis of the repeat copy numbers (RCNs) and analysis of the coefficient of variability (CV) of the P-SSRs were analyzed according to the methods described by Qi et al. (2020), which was able to reveal the degree of variation in the RCNs of different SSRs.

### Functional Annotation of the P-SSRs

To characterize the functional roles of the CDSs containing P-SSRs, these sequences were aligned with the NCBI non-redundant database and the SWISS-PROT database using Diamond (Buchfink et al., 2015) with a cutoff E-value of 1E-5. Gene Ontology (GO) term mapping was conducted by TBtools. The mapping results were submitted to WEGO (Ye et al., 2018) for GO classification, and TBtools was further used to perform GO enrichment analysis. Kyoto Encyclopedia of Genes and Genomes (KEGG) pathway analysis was carried out by a KEGG automatic annotation server called KAAS (KEGG Automatic Annotation Server^2^; Moriya et al., 2007). The output contains KO (KEGG Orthology) assignments that were used for KEGG enrichment analysis by TBtools.

### Statistical Analysis

SPSS (Statistical Product and Service Solutions, version 17.0) was used for the calculation of the Pearson correlation coefficient and the significance test. Figures were produced using Microsoft Office Excel 2013, ImageGP^3^, and R (version 3.5.1) with the “ggplot2” package.

## Results

### Occurrence of SSRs in Euarchontoglires Genomes

The basic attributes of different SSR categories derived from our analysis along with the taxonomic classification of each organism are presented in Supplementary Table 2. A total of 702,828,080 SSR loci (P-SSRs, C-SSRs, and I-SSRs) were identified from the genome data of 153 species. The length proportions of the SSRs covered from 3.19% (*Daubentonia madagascariensis*) to 9.87% (*Myocastor coypus*) of the Euarchontoglires genomes. Of these, I-SSRs were the dominant SSR category (62.05–82.58%) of the SSRs recovered, followed by P-SSRs (16.77–34.64%), and C-SSRs (0.60–4.17%). Supplementary Table 3 shows that both the total number and length of SSRs (including P-SSRs, C-SSRs, and I-SSRs) were positively correlated with genome size (SSR numbers: Pearson, *r* = 0.619, *p* < 0.01; SSR length: Pearson, *r* = 0.495, *p* < 0.01). Moreover, the total number and total length of the SSRs in rodents, primates, lagomorphs, and tree shrews were positively correlated with genome size (Supplementary Table 3). However, the abundance and density of SSRs were not significantly correlated with the genome size of Euarchontoglires (SSR abundance: Pearson, *r* = 0.092, *p* = 0.534; SSR density: Pearson, *r* = 0.028, *p* = 0.73). Although the abundance and density of SSRs in rodent genomes were not significantly correlated with genome size (SSR abundance: Pearson, *r* = 0.082, *p* = 0.449; SSR density: Pearson, *r* = 0.068, *p* = 0.53), the abundance and density of SSRs in primate genomes were significantly positively correlated with genome size (SSR abundance: Pearson, *r* = 0.534, *p* < 0.001; SSR density: Pearson, *r* = 0.528, *p* < 0.001). Furthermore, no significant relationship between genome size and SSR abundance or SSR density was found in lagomorphs and tree shrews. In the genic regions, the abundance of SSRs also followed the pattern I-SSRs > P-SSRs > C-SSRs (Supplementary Table 4). Moreover, the abundance of SSRs in different genic regions followed the pattern intron > exon > CDS.

### Variation Characteristics of P-SSRs Across the Evolutionary Landscape

**FIGURE 1:**
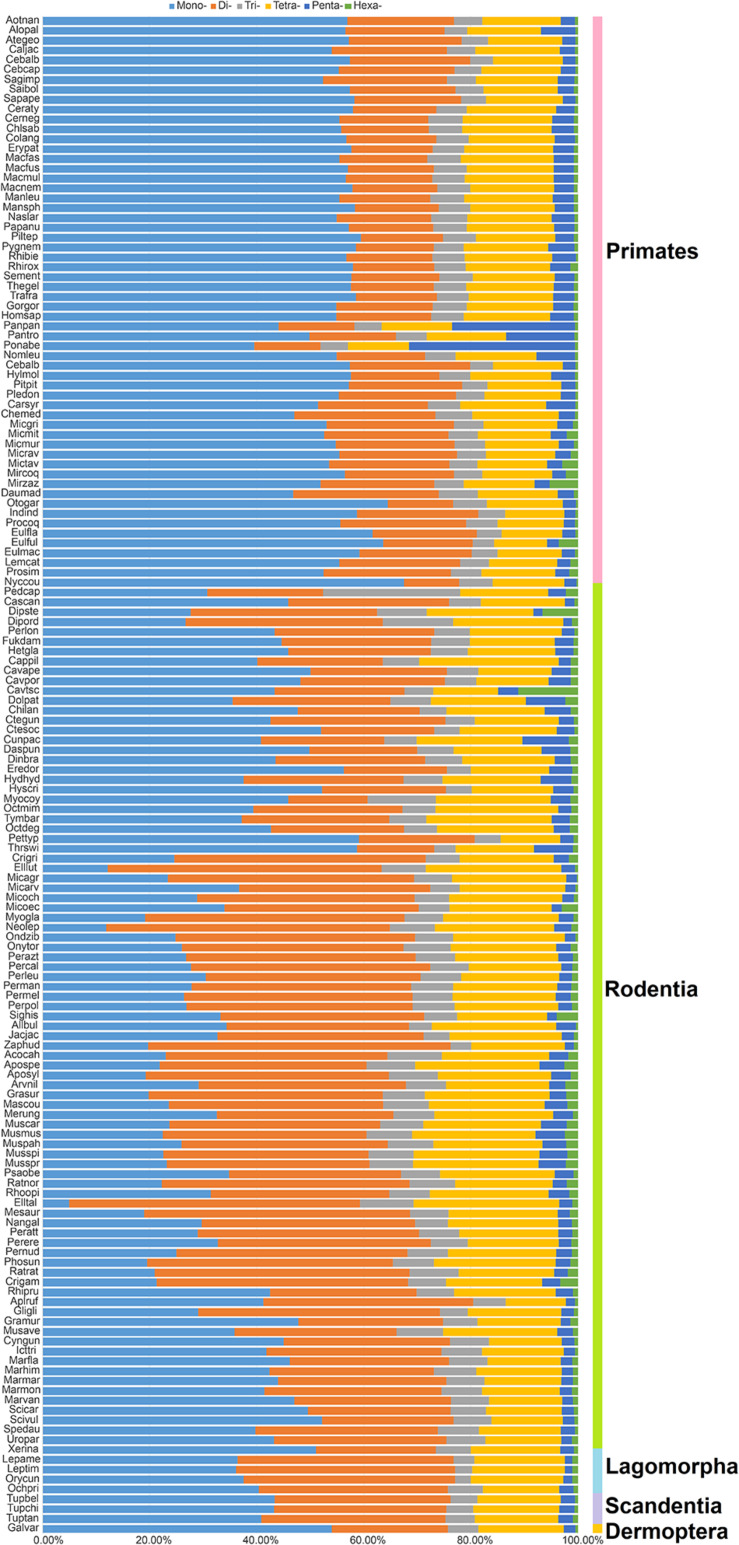
Percentage of six categories of P-SSRs in the 153 Euarchontoglires genomes. Percentages were calculated according to the total number of each P-SSR type divided by the total number of P-SSRs in that species.

As presented in Figure 1, the most frequent categories of P-SSRs in primates, tree shrews, and colugo were identical (i.e., mononucleotide P-SSRs), while mono- or dinucleotide motif types were the most abundant P-SSRs in both rodents and lagomorphs. In rodents, SSR abundance varied among the studied genomes. For example, the dominant P-SSR category tends to be conserved within most genera, such as *Peromyscus*, *Mus, Rattus*, or *Cavia* (Figure 1). In the *Microtus* genus (4 species), however, dinucleotide P-SSRs dominated in *Microtus agrestis*, *Microtus ochrogaster*, and *Microtus oeconomus* but not in *Microtus arvalis*, in which the proportion of mononucleotide P-SSRs (36.74%) was slightly higher than that of dinucleotide P-SSRs (35.69%). Next, the numbers of P-SSRs in the CDS, exon, and intron regions of the Euarchontoglires genomes were further analyzed (Table 1). As expected, trinucleotide P-SSRs dominated in the CDS regions of all species analyzed in this study. Similarly, the most prevalent P-SSR categories in the exon and intron regions of primates, tree shrews, and colugos were mononucleotide P-SSRs, while the most common P-SSR types in rodents and lagomorphs were mono- or dinucleotide P-SSRs. The predominant P-SSR type in most rodents within the same genus was conserved in the exon and intron regions, but the dominant P-SSR type in the exon regions varied among species within *Mus* (Table 1).

**TABLE 1:** The proportion of mono- to hexanucleotide P-SSRs in genic regions.

Order	Suborder	Family	Species	CDS						Exon						Intron					
				Mono-	Di-	Tri-	Tetra-	Penta-	Hexa-	Mono-	Di-	Tri-	Tetra-	Penta-	Hexa-	Mono-	Di-	Tri-	Tetra-	Penta-	Hexa-
Primates	Haplorrhini	Aotidae	*Aotus nancymaae*	2.51%	2.17%	88.69%*	1.66%	0.57%	4.40%	55.23%*	19.90%	13.74%	8.33%	2.09%	0.715%	60.88%*	18.01%	4.78%	13.28%	2.50%	0.55%
	Haplorrhini	Cebidae	*Saimiri boliviensis*	4.94%	3.05%	84.01%*	1.89%	0.51%	5.60%	62.65%*	17.56%	10.20%	6.28%	2.41%	0.91%	61.61%*	17.36%	4.75%	12.69%	2.86%	0.72%
	Haplorrhini	Cebidae	*Sapajus apella*	3.21%	2.07%	88.60%*	1.43%	0.30%	4.39%	58.88%*	18.47%	12.50%	7.49%	1.99%	0.69%	62.52%*	17.95%	4.13%	12.71%	2.27%	0.42%
	Haplorrhini	Cercopithecidae	*Cercocebus atys*	22.90%	5.14%	63.97%*	4.47%	0.58%	2.94%	56.01%*	18.46%	12.59%	9.30%	2.89%	0.75%	61.52%*	14.27%	5.60%	14.69%	3.31%	0.61%
	Haplorrhini	Cercopithecidae	*Chlorocebus sabaeus*	4.38%	4.97%	83.40%*	2.44%	1.22%	3.58%	55.58%*	19.59%	11.13%	9.68%	3.20%	0.82%	59.81%*	14.81%	5.67%	15.02%	3.98%	0.71%
	Haplorrhini	Cercopithecidae	*Colobus angolensis*	2.81%	2.04%	88.95%*	1.90%	0.35%	3.94%	61.00%*	17.71%	10.63%	7.37%	2.60%	0.69%	61.11%*	14.90%	5.56%	14.41%	3.48%	0.53%
	Haplorrhini	Cercopithecidae	*Macaca mulatta*	1.50%	1.86%	90.27%*	1.99%	0.58%	3.81%	55.72%*	17.88%	13.56%	9.06%	2.98%	0.80%	60.86%*	14.55%	5.51%	14.85%	3.62%	0.62%
	Haplorrhini	Cercopithecidae	*Macaca nemestrina*	2.48%	3.05%	87.97%*	2.29%	0.53%	3.68%	54.93%*	18.81%	12.82%	9.52%	3.04%	0.88%	60.87%*	14.91%	5.55%	14.45%	3.56%	0.66%
	Haplorrhini	Cercopithecidae	*Mandrillus leucophaeus*	2.84%	3.19%	87.79%*	1.66%	0.49%	4.02%	62.24%*	16.27%	10.33%	7.49%	2.78%	0.88%	60.15%*	14.96%	5.67%	14.76%	3.78%	0.68%
	Haplorrhini	Cercopithecidae	*Papio anubis*	3.11%	3.02%	87.88%*	1.74%	0.46%	3.80%	54.53%*	20.34%	11.32%	10.09%	2.97%	0.76%	61.27%*	14.33%	5.56%	14.56%	3.67%	0.62%
	Haplorrhini	Cercopithecidae	*Piliocolobus tephrosceles*	22.11%	1.61%	71.44%*	0.81%	0.20%	3.83%	61.92%*	16.18%	11.73%	7.25%	2.08%	0.84%	63.03%*	13.82%	5.29%	13.88%	3.42%	0.56%
	Haplorrhini	Cercopithecidae	*Rhinopithecus bieti*	3.07%	2.49%	88.61%*	1.68%	0.58%	3.59%	54.22%*	18.58%	13.78%	8.81%	3.98%	0.63%	61.86%*	13.90%	5.45%	14.35%	4.18%	0.26%
	Haplorrhini	Cercopithecidae	*Rhinopithecus roxellana*	1.95%	1.57%	90.56%*	1.76%	0.33%	3.81%	57.67%*	15.47%	15.38%	7.64%	2.86%	0.97%	62.89%*	13.45%	5.27%	14.22%	3.54%	0.63%
	Haplorrhini	Cercopithecidae	*Theropithecus gelada*	2.27%	1.93%	90.16%*	1.40%	0.24%	4.00%	58.71%*	14.88%	15.53%	7.59%	2.47%	0.82%	62.34%*	13.72%	5.34%	14.28%	3.66%	0.65%
	Haplorrhini	Cercopithecidae	*Trachypithecus francoisi*	2.31%	2.26%	89.50%*	1.46%	0.66%	3.81%	56.70%*	16.52%	14.95%	8.35%	2.70%	0.79%	63.17%*	13.42%	5.34%	14.12%	3.35%	0.61%
	Haplorrhini	Hominidae	*Gorilla gorilla*	1.29%	1.93%	90.85%*	1.48%	0.49%	3.96%	53.81%*	17.32%	15.86%	9.06%	2.97%	0.98%	59.76%*	15.85%	5.65%	14.59%	3.46%	0.70%
	Haplorrhini	Hominidae	*Homo sapiens*	1.74%	1.69%	90.25%*	2.60%	0.45%	3.26%	54.54%*	18.65%	11.44%	11.21%	3.18%	0.97%	58.40%*	16.74%	5.61%	15.02%	3.52%	0.71%
	Haplorrhini	Hominidae	*Pan paniscus*	4.33%	1.93%	86.46%*	3.11%	0.88%	3.28%	59.31%*	15.15%	11.32%	10.13%	3.28%	0.82%	58.56%*	16.25%	5.77%	14.99%	3.80%	0.63%
	Haplorrhini	Hominidae	*Pan troglodytes*	2.87%	3.18%	87.16%*	2.03%	1.10%	3.66%	52.61%*	19.73%	13.33%	10.45%	3.10%	0.78%	59.01%*	16.03%	5.74%	15.01%	3.59%	0.61%
	Haplorrhini	Hominidae	*Pongo abelii*	1.46%	1.99%	90.00%*	1.55%	0.58%	4.42%	55.03%*	16.04%	16.95%	8.48%	2.66%	0.84%	60.07%*	15.45%	6.22%	14.37%	3.24%	0.64%
	Haplorrhini	Hylobatidae	*Nomascus leucogenys*	2.15%	1.77%	90.11%*	1.68%	0.47%	3.82%	55.37%*	16.93%	16.14%	7.87%	2.92%	0.78%	61.90%*	15.12%	5.34%	13.93%	3.20%	0.50%
	Haplorrhini	Hylobatidae	*Hylobates moloch*	3.00%	2.47%	88.34%*	1.76%	0.57%	3.85%	56.44%*	17.27%	14.88%	7.88%	2.81%	0.72%	62.73%*	14.74%	5.23%	13.61%	3.18%	0.51%
	Haplorrhini	Tarsiidae	*CarlitoCarlit syrichta*	3.47%	2.45%	85.70%*	1.78%	0.68%	5.92%	55.28%*	20.19%	14.58%	6.64%	2.27%	1.03%	60.08%*	17.74%	5.01%	13.76%	3.00%	0.40%
	Strepsirrhini	Cheirogaleidae	*Microcebus murinus*	2.34%	1.69%	89.56%	1.92%	0.27%	4.21%	50.85%*	21.17%	15.27%	10.00%	2.23%	0.47%	58.89%*	20.09%	4.89%	12.89%	2.80%	0.44%
	Strepsirrhini	Galagidae	*Otolemur garnettii*	2.39%	1.55%	89.18%*	2.60%	0.35%	3.94%	57.55%*	14.42%	17.19%	8.01%	2.14%	0.69%	69.01%*	10.20%	5.70%	12.56%	2.24%	0.30%
	Strepsirrhini	Indriidae	*Propithecus coquereli*	1.55%	1.44%	91.58%*	1.39%	0.16%	3.89%	50.41%*	15.17%	24.39%	7.77%	1.69%	0.56%	61.38%*	20.04%	4.88%	11.35%	1.97%	0.38%
Rodentia	Castorimorpha	Castoridae	*Castor canadensis*	1.62%	1.91%	88.41%*	2.49%	0.81%	4.75%	53.05%*	25.01%	5.62%	14.06%	1.88%	0.40%	53.53%*	25.26%	4.75%	14.24%	1.87%	0.36%
	Castorimorpha	Heteromyidae	*Dipodomys ordii*	1.77%	1.82%	90.69%*	1.93%	0.48%	3.32%	34.77%	36.04%* 8 *	8.54%	18.60%	1.65%	0.40%	34.85%	36.27%*	8.08%	18.77%	1.65%	0.38%
	Hystricomorpha	Bathyergidae	*Fukomys damarensis*	2.26%	3.63%	84.84%*	2.66%	0.48%	6.13%	44.77%*	26.83%	12.81%	11.33%	3.41%	0.84%	48.97%*	24.79%	6.43%	15.43%	3.61%	0.76%
	Hystricomorpha	Bathyergidae	*Heterocephalus glaber*	3.01%	2.34%	85.79%*	1.78%	1.06%	6.02%	49.24%*	21.19%	12.79%	12.09%	3.59%	1.09%	51.16%*	23.20%	6.00%	15.43%	3.49%	0.72%
	Hystricomorpha	Caviidae	*Cavia porcellus*	2.32%	1.84%	84.59%*	2.11%	1.09%	8.04%	50.34%*	21.86%	13.30%	9.19%	3.67%	1.63%	54.37%*	22.68%	5.00%	13.24%	3.70%	1.01%
	Hystricomorpha	Chinchillidae	*Chinchilla lanigera*	1.92%	1.47%	85.60%*	2.50%	0.64%	7.87%	52.06%*	21.75%	11.00%	10.11%	3.91%	1.18%	51.43%*	20.70%	4.46%	17.52%	4.72%	1.17%
	Hystricomorpha	Octodontidae	*Octodon degus*	1.29%	1.60%	87.29%*	1.96%	0.31%	7.55%	51.17%*	17.37%	18.61%	8.78%	2.63%	1.43%	48.43%*	22.70%	5.19%	19.61%	2.84%	1.22%
	Myomorpha	Cricetidae	*Cricetulus griseus*	4.49%	3.18%	82.12%*	2.78%	0.90%	6.53%	44.36%*	30.07%	8.13%	13.17%	3.17%	1.11%	28.77%	42.90%*	5.99%	17.93%	2.94%	1.47%
	Myomorpha	Cricetidae	*Microtus ochrogaster*	1.29%	1.53%	92.51%*	1.13%	0.16%	3.38%	47.09%*	25.41%	12.05%	12.40%	2.55%	0.50%	31.43%	38.39%*	6.00%	21.25%	2.26%	0.67%
	Myomorpha	Cricetidae	*Neotoma lepida*	1.09%	2.69%	89.44%*	2.69%	0.95%	3.13%	15.53%	48.36%*	8.87%	22.65%	3.45%	1.14%	15.53%	48.36%*	8.87%	22.65%	3.45%	1.14%
	Myomorpha	Cricetidae	*Peromyscus leucopus*	1.60%	0.95%	92.59%*	1.60%	0.39%	2.86%	42.46%*	24.22%	18.38%	11.77%	2.49%	0.68%	32.48%	38.69%*	7.14%	18.39%	2.39%	0.91%
	Myomorpha	Cricetidae	*Peromyscus maniculatus*	2.08%	1.44%	91.14%*	1.34%	0.54%	3.47%	43.98%*	24.26%	15.72%	12.34%	2.89%	0.81%	26.61%	46.91%*	6.45%	16.78%	2.26%	0.98%
	Myomorpha	Dipodidae	*Jaculus jaculus*	2.39%	1.53%	89.73%*	2.08%	0.86%	3.41%	46.91%*	12.93%	27.11%	8.87%	2.56%	1.62%	34.63%	36.29%*	4.40%	21.61%	2.40%	0.66%
	Myomorpha	Muridae	*Arvicanthis niloticus*	4.42%	1.62%	85.66%*	1.35%	0.11%	6.85%	49.78%*	21.45%	12.74%	11.85%	3.07%	1.12%	31.23%	37.43%*	6.59%	19.88%	3.04%	1.83%
	Myomorpha	Muridae	*Grammomys surdaster*	1.79%	1.46%	90.45%*	1.92%	0.33%	4.05%	42.77%*	26.38%	14.61%	12.45%	2.76%	1.03%	23.38%	41.10%*	6.94%	23.64%	3.14%	1.80%
	Myomorpha	Muridae	*Mastomys coucha*	0.82%	1.87%	89.72%*	2.42%	0.38%	4.78%	36.01%*	28.80%	15.23%	14.35%	4.42%	1.20%	25.80%	38.75%*	7.75%	21.76%	4.19%	1.75%
	Myomorpha	Muridae	*Meriones unguiculatus*	1.68%	1.22%	92.16%*	1.31%	0.32%	3.31%	49.83%*	19.31%	16.24%	10.99%	3.18%	0.46%	35.16%*	31.91%	6.54%	21.91%	3.69%	0.78%
	Myomorpha	Muridae	*Mus caroli*	1.90%	2.38%	90.07%*	1.49%	0.36%	3.80%	39.48%*	25.73%	15.12%	14.21%	4.41%	1.05%	26.23%	38.16%*	7.73%	21.48%	4.64%	1.75%
	Myomorpha	Muridae	*Mus musculus*	2.24%	4.02%	83.54%*	3.37%	0.75%	6.08%	31.61%	33.03%*	10.49%	18.49%	4.84%	1.54%	23.52%	38.42%*	7.84%	22.78%	5.22%	2.23%
	Myomorpha	Muridae	*Mus pahari*	1.77%	1.50%	90.08%*	1.44%	0.17%	5.04%	44.15%*	23.22%	15.19%	11.98%	4.27%	1.19%	28.45%	37.23%*	7.77%	20.21%	4.38%	1.96%
	Myomorpha	Muridae	*Rattus norvegicus*	3.01%	3.12%	84.99%*	2.41%	0.60%	5.86%	39.31%*	30.84%	10.12%	14.70%	3.73%	1.31%	24.82%	44.49%*	7.57%	18.59%	2.68%	1.86%
	Myomorpha	Muroidea	*Mesocricetus auratus*	1.13%	1.45%	91.81%*	1.51%	0.31%	3.78%	29.57%	35.81%*	15.12%	15.54%	3.12%	0.85%	22.29%	46.89%*	6.53%	20.76%	2.26%	1.26%
	Myomorpha	Muroidea	*Nannospalax galili*	2.31%	1.75%	88.93%*	2.69%	0.25%	4.07%	47.86%*	25.15%	13.87%	9.68%	2.67%	0.76%	35.24%	36.55%*	5.59%	19.19%	2.59%	0.83%
	Myomorpha	Muroidea	*Rattus rattus*	2.24%	1.59%	90.14%*	1.42%	0.18%	4.43%	43.46%*	23.78%	15.68%	12.14%	3.81%	1.14%	23.32%	46.22%*	8.12%	18.15%	2.52%	1.67%
	Sciuromorpha	Sciuridae	*Ictidomys tridecemlineatus*	2.06%	1.56%	90.22%*	1.35%	0.14%	4.68%	58.38%*	19.03%	11.24%	8.02%	2.89%	0.43%	46.72%*	29.34%	6.64%	14.89%	2.00%	0.42%
	Sciuromorpha	Sciuridae	*Marmota flaviventris*	2.02%	1.36%	91.12%*	0.98%	0.27%	4.25%	59.10%*	16.13%	15.47%	6.80%	1.98%	0.51%	51.66%*	26.50%	6.17%	13.43%	1.86%	0.38%
	Sciuromorpha	Sciuridae	*Urocitellus parryii*	1.53%	0.60%	90.84%*	0.98%	0.49%	5.56%	48.28%*	28.66%	6.77%	13.90%	1.91%	0.48%	48.42%*	28.95%	6.23%	14.05%	1.90%	0.45%
Lagomorpha		Leporidae	*Oryctolagus cuniculus*	2.78%	2.04%	88.03%*	1.30%	0.46%	5.38%	2.69%	45.12%*	28.23%	16.14%	4.87%	2.95%	40.70%*	36.27%	2.67%	17.60%	1.74%	1.02%
		Ochotonidae	*Ochotona princeps*	1.38%	1.44%	93.46%*	0.96%	0.36%	2.40%	57.15%*	15.63%	17.24%	7.32%	2.49%	0.17%	44.43%*	32.69%	6.60%	13.27%	2.56%	0.45%
Dermoptera		Cynocephalidae	*Galeopterus variegatus*	2.75%	2.12%	89.90%*	1.91%	0.49%	2.82%	60.22%*	17.34%	12.19%	7.69%	2.04%	0.52%	62.30%*	17.77%	4.58%	13.01%	1.98%	0.36%
Scandentia		Tupaiidae	*Tupaia chinensis*	6.55%	2.55%	82.55%*	1.36%	1.02%	5.96%	55.44%*	21.75%	9.59%	8.91%	3.26%	1.05%	45.50%*	30.97%	4.66%	15.63%	2.70%	0.54%

*the dominant P-SSR type.

**FIGURE 2:**
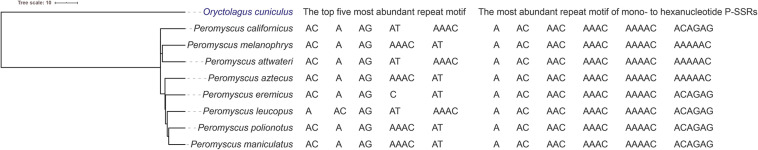
The top five most abundant repeat motifs and the most common repeat motif in each P-SSR category in the *Peromyscus* genus. The phylogenetic tree was derived from TimeTree (http://www.timetree.org/), and *Oryctolagus cuniculus* was used as the outgroup. *Peromyscus nudipes* could not be found in Timetree and therefore is not shown in this tree.

The top five most abundant repeat motifs and the most dominant repeat motifs with different lengths are shown in Supplementary Table 5. Our results demonstrated that the most predominant P-SSR motifs showed some taxon-specific features. (A)n was the most recurrent motif in primates, lagomorphs, tree shrews, and colugo, while (A)n, (AC)n, or (AG)n was the most abundant motif in rodent genomes. Furthermore, the most dominant SSR motifs within some genera in Rodentia varied (e.g., *Peromyscus*). On the other hand, the results showed that the most frequent repeated motif in each P-SSR category (mono- to hexanucleotide P-SSRs) exhibited different levels of variation among the mammals studied. Moreover, such variation was greater in rodents than in primates (Supplementary Table 5). For the most frequent repeat motifs of mononucleotide P-SSRs, (A)n dominated in all Euarchontoglires genomes. The dominant dinucleotide repeat motif was (AC)n in primates and colugos. The most frequent dinucleotide motif in rodent and lagomorph genomes was (AC)n and (AG)n, whereas (AG)n repeats rarely occurred. The most frequent dinucleotide motif in tree shrews was (AG)n. Additionally, (AAT)n and (AAC)n dominated in primate and lagomorph genomes; (AAT)n, (AAC)n, and (AGG)n were the most common trinucleotide repeat motifs; and (AAT)n was the most dominant motif in tree shrews and colugos. For tetranucleotide P-SSRs, the predominant motif in the genomes of primates, lagomorphs, tree shrews, and colugo was (AAAT)n, while the predominant motifs of tetranucleotides in rodents were (AAAG)n, (AAAT)n, and (AAAC)n. However, the predominant motifs of penta- or hexanucleotide P-SSRs differed considerably among these Euarchontoglires genomes. It is noteworthy that the dominant repeat motif might differ among species belonging to the same genus in different clades, for instance, the dominant hexanucleotide P-SSR motifs in *Peromyscus* (Supplementary Table 5). However, the similarity of the dominant motif among species in the same genus did not coincide with the topological structure of the tree (Figure 2).

Although the dominant P-SSR repeat motif in genic regions (especially in CDS regions) varied within Euarchontoglires, a few taxon-specific features were observed (Supplementary Table 5). For example, (C)n was the most frequent mononucleotide P-SSR motif in genic regions (CDS, exon, and intron regions) of all Euarchontoglires genomes (Supplementary Table 5). Furthermore, (AC)n was the most abundant dinucleotide P-SSR motif in intron regions of primates and colugo; (AC)n or (AG)n was the most frequent motifs in rodents and lagomorphs; and (AG)n was dominant in tree shrews. Notably, the dominant triplet P-SSR motif types in the CDS regions showed more diversity than those in the exon and intron regions. For example, (AAAAAC)n was dominant in intron regions of most primates, while striking variability of the dominant hexanucleotide repeat motif was observed in the CDS regions of rodent genomes. Nevertheless, the most dominant trinucleotide repeat motifs in the CDS regions of all genomes were (ACG)n, (AGG)n, (CCG)n, and (AGG)n.

**FIGURE 3:**
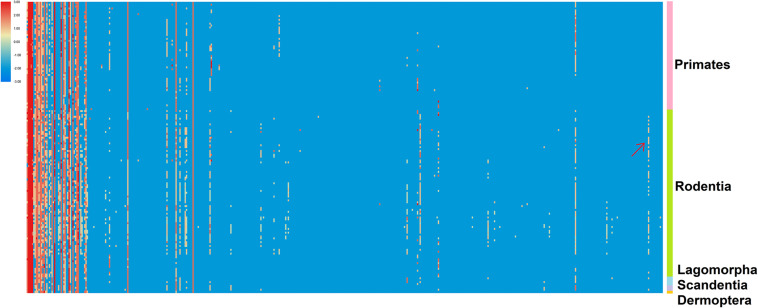
The enrichment trend of the 501 P-SSRs across 153 genomes. The P-SSR density-based ranking is used to generate a heat map per the color scale indicated. The 501 P-SSR motifs are arranged in columns, and the 153 organisms are arranged in rows. Arrows mark the positions for uniquely abundant (enriched) SSR signatures described in the text.

As described by Srivastava et al. (2019), a ranked P-SSR density heat map was plotted to illustrate the density-based abundance trends of the 501 SSRs (columns) across all 153 genomes (rows) in our study (Figure 3 and Supplementary Table 6). Our results demonstrated that some clear patterns of abundance that were distinct for different subgroups of Euarchontoglires could be detected in the heat map. As seen along the left-most columns of the Figure 3, a few P-SSR motifs are highly abundant across most organisms, such as A, AC, AG, AAAT, AAAG, and other polyA repeat classes. Moreover, the density of some motif types was relatively higher in specific groups, but relatively rare in other groups. For example, the density of (AAGAGG)n was relatively higher in rodents than that in other clades, as indicated by arrow in Figure 3. Furthermore, comparison of microsatellite motifs demonstrated that clade-specific motifs were only found in rodents: (AAGCGT)n, (ATCGCG)n, (AACGGT)n, (AACGTC)n, (AACGAT)n, (ATGCTC)n, (AGCTCG)n, and (AAACGT)n. However, such motifs were only shared by several rodent species. For example, (AACGTC)n was shared by four rodent species, while (AAGCGT)n was only found in *Peromyscus maniculatus*. The specific repeat motifs in genic and intergenic regions of different clades were further analyzed (not shown), which revealed that the number of the specific repeat motif categories in rodents was higher than that in other clades of Euarchontoglires.

### Analysis of the Coefficient of Variability of the P-SSRs

**FIGURE 4:**
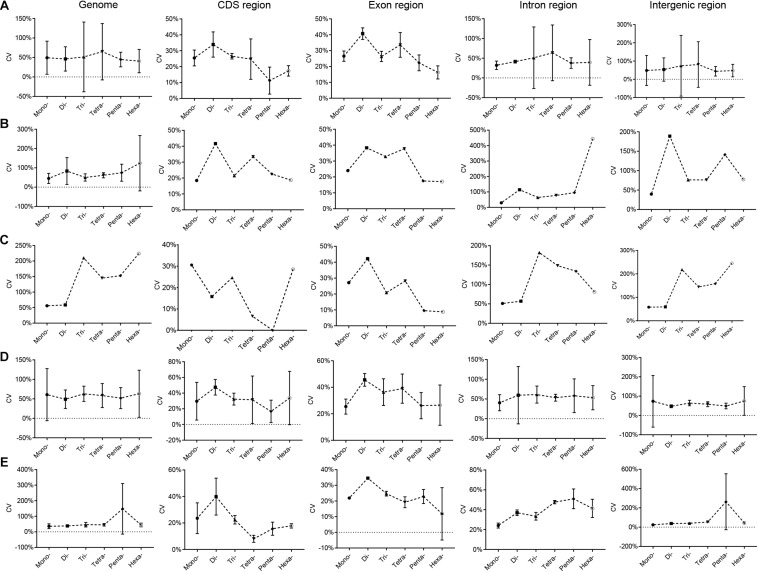
CV analysis of the RCNs of P-SSRs in Euarchontoglires genomes. **(A)**, Primates; **(B)**, Scandentia; **(C)**, Dermoptera; **(D)**, Rodentia; **(E)**, Lagomorpha.

The variation characteristics of RCNs of different SSR types in Euarchontoglires genomes are shown in Supplementary Table 7. The CV of the RCN of P-SSRs with the same motif length (e.g., mononucleotide P-SSRs) varied differentially among the species in Euarchontoglires. In *Peromyscus* genus, for example, the CV of the RCN of the hexanucleotide P-SSR in genomes varied from 30.39% (*Peromyscus nudipes*) to 343.14% (*Peromyscus eremicus*). Furthermore, the trend line for the CV of the mono- to hexanucleotide P-SSRs among different species also differed considerably (Supplementary Table 8). Nonetheless, a few common characteristics of the CV of the RCN of P-SSRs were detected in the CDS and exon regions. For example, the CVs of tetra- and pentanucleotide P-SSRs were relatively lower compared with those of trinucleotide P-SSRs in most species. Moreover, the mean CV of the mono- to hexanucleotide P-SSR among the five subclades of Euarchontoglires exhibited comparable trends (Figure 4). From di- to hexanucleotide P-SSRs in exon regions, for example, the CV decreased as the P-SSR motif length increased. Furthermore, the patterns of CV of P-SSRs in genomes showed similar pattern to that in intergenic regions, while the CV of P-SSRs in CDS and exon regions showed similar pattern.

### GC Content of P-SSRs in Euarchontoglires Genomes

**FIGURE 5:**
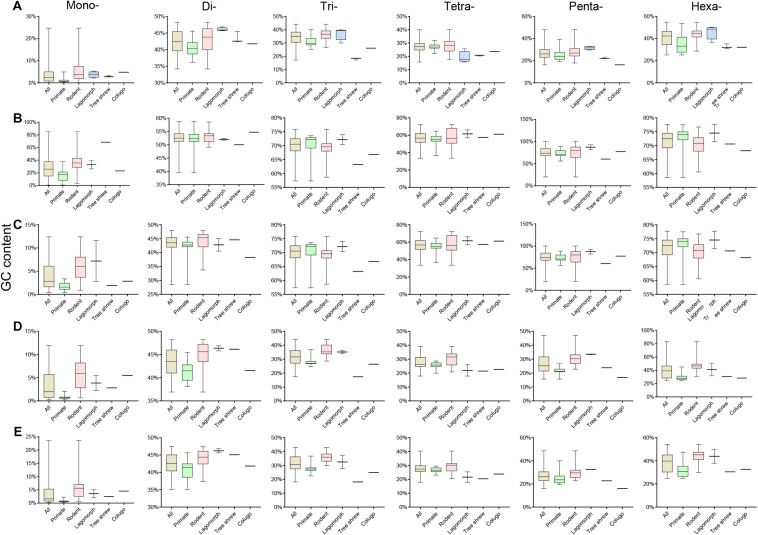
The GC contents of P-SSRs in genomes and in genomic regions of different taxon clades within Euarchontoglires. **(A)**, genomes; **(B)**, CDSs; **(C)**, exons; **(D)**, introns; **(E)**, intergenic regions.

Assessment of the GC content variation of P-SSRs in different subgroups of Euarchontoglires was also performed in this study (Figure 5 and Supplementary Table 9), which demonstrated that the P-SSR categories containing the highest or the lowest GC were relatively conserved. As shown in Figure 5 and Supplementary Table 9, mononucleotide P-SSRs had the lowest GC content in all genomes studied, while dinucleotide or hexanucleotide P-SSRs had the highest GC content across most genomes. However, different levels of GC content variation could be observed in different clades when compared at different taxonomic levels. The P-SSR category that had the highest GC content varied within some genera; for example, mononucleotide P-SSRs in *Eulemur flavifrons* and *Eulemur macaco* had the highest GC content, whereas hexanucleotide P-SSRs had the highest GC content in *Eulemur fulvus*. This pattern in GC content was not consistent with the topological structure of the evolutionary relationships within *Eulemur*. In genic regions, the P-SSR category containing the highest GC content in the exon and intron regions was relatively conserved in primates compared with rodents (Supplementary Table 9). For example, trinucleotide P-SSRs had the highest GC content in exon regions of most primates (except for *Rhinopithecus bieti*), while di- (12 species) or hexanucleotide P-SSRs (15 species) had the highest GC content in rodents (Supplementary Table 9). In addition, the GC content was higher for tetra-, penta-, or hexanucleotide P-SSRs than for other P-SSR categories, which was caused by the small amount of tetra- to hexanucleotide P-SSRs in the CDS regions. In intergenic regions, the P-SSR category with the lowest GC content was mononucleotide P-SSRs in most Euarchontoglires species. Furthermore, the GC content of mono- to hexanucleotide P-SSRs in most rodents was higher than that in primates.

### Functional Analysis of CDSs With P-SSRs in the Genomes of Euarchontoglires

**FIGURE 6:**
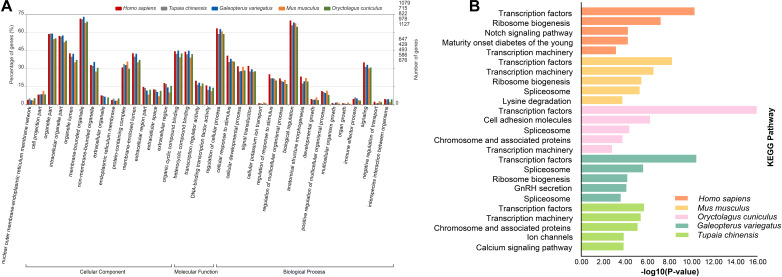
The results of GO **(A)** and KEGG **(B)** analysis for the CDSs containing SSRs in genomes of five representatives within Euarchontoglires (*Homo sapiens*, *Tupaia chinensis*, *Galeopterus variegatus*, *Mus musculus*, and *Oryctolagus cuniculus*).

To characterize the functional roles of the CDSs possessing P-SSRs, we performed GO and KEGG pathway enrichment analyses. Surprisingly, GO and KEGG pathway enrichment analyses for the CDS containing P-SSRs in different clades of Euarchontoglires obtained similar results (Supplementary Table 10, Supplementary Table 11). As shown in Supplementary Table 10, the molecular function (MF) category of the GO analysis showed that the CDSs containing P-SSRs in all genomes were significantly enriched in “binding” and “transcription regulator activity.” For the biological process (BP) category of the GO analysis, these CDSs were mainly associated with developmental process, immune system process, and metabolic process. Furthermore, these sequences were involved with “cell,” “intracellular,” or “protein-containing complex” in the cellular component (CC) categories. Supplementary Table 11 shows the results of the KEGG analysis, and Figure 6 shows the KEGG analysis results of *Homo sapiens*, *Mus musculus*, *Oryctolagus cuniculus*, *Tupaia chinensis*, and *Galeopterus variegatus*. The CDSs containing P-SSRs in the genomes of *Homo sapiens*, *Mus musculus*, *Oryctolagus cuniculus*, *Tupaia chinensis*, and *Galeopterus variegatus* were mainly enriched in transcription factors (Figure 6B).

Transcription factors, which bind preferentially to certain DNA sequences, play the central role of transcriptional regulation in all organisms (Imlay, 2015; Symonenko et al., 2018; Mejhert et al., 2020). In this study, the transcription factors containing SSRs were further identified from the annotation files and the results in Supplementary Table 11 by a Python script. Our results revealed that the most abundant transcription factors containing P-SSRs in different clades of Euarchontoglires were zinc finger protein and forkhead-box protein (Supplementary Table 12).

## Discussion

### The Distribution Patterns of SSRs in Euarchontoglires Genomes

Although the contributions of SSRs to variation in genome size remain unclear, the genome size variation among eukaryotic species is more closely correlated with the amount of repetitive DNA rather than the number of coding genes (Bennetzen et al., 2005; Blommaert et al., 2019). Our results showed that the number and length of SSRs were positively correlated with genome size, which is consistent with previous studies (Zhao et al., 2012; Qi et al., 2016; Ding et al., 2017). However, differences in the relationships between genome size and SSR abundance or SSR density were observed for primates and rodents. Our findings revealed a significant positive relationship between genome size and SSR abundance or density in primates, but no significant relationship was observed for rodents (Supplementary Table 3). Likewise, variable results have been derived from different taxon groups. For example, a negative relationship between genome size and SSR abundance has been observed in insects (Ding et al., 2017), but no correlation or no significant relationship has been observed in fungi (Wang et al., 2014), birds (Huang et al., 2016), or primates (Xu et al., 2018). In addition to the SSR detection criteria and sampling size, we suggested that the relationship between genome size and SSR density (or SSR abundance) might differ in different clades. Indeed, our results supported this hypothesis. Moreover, a significant positive relationship between SSR abundance (or density) and the genome size of species in Euarchontoglires was observed. Consequently, such a relationship (e.g., the relationship between SSR abundance and genome size) derived from a higher taxonomic level might not hold within its subgroups.

The proportion of different SSR categories was highly conserved in genomes or in genic regions across species in Euarchontoglires and showed the pattern I-SSRs > P-SSRs > C-SSRs (Supplementary Tables 2, 4), which is consistent with patterns observed in beetles that used the same SSR detection criteria (Song et al., 2020). Better insight into the occurrence of SSRs in a range of taxa under an evolutionary scenario is important for understanding the differential abundance of SSRs. It remains to be seen whether the prevalence of I-SSR is common in other lineages with the same SSR detection criteria. Changes in motif units by insertions, substitutions, and deletions of nucleotides produce I-SSRs and C-SSRs, which show decreased mutation rates compared with P-SSRs (Sainudiin et al., 2004). The dominance of I-SSRs in CDS regions has been suggested to play an important role in preventing coding-region frameshifts induced by microsatellite instability (Song et al., 2016). Therefore, the prevalence of I-SSRs in the genome might reflect the important role that the DNA repair system plays in the regulation of microsatellite instability. Moreover, the I-SSRs concentrated at certain locus in potyvirus genomes (e.g., HC-Pro helper component proteinase and coat protein) could be involved in recombination, producing genetic variation that drives host adaptation (Alam et al., 2013). Additional study of the dominance of I-SSRs in genomes may be useful for better understanding of genetic variation in Euarchontoglires species.

### Taxon-Specific Features of P-SSRs in Euarchontoglires Genomes

Taxon-specific P-SSR distribution patterns have been detected in some lineages of organisms (Qin et al., 2015; Ding et al., 2017; Srivastava et al., 2019). However, few studies have examined variation in the distribution of microsatellites in Euarchontoglires at different taxonomic levels. Despite the controversial placement of Scandentia (Kumar et al., 2013), the most abundant P-SSR category in the genomes of the primate, tree shrew, and colugo clade (mononucleotide P-SSR) was different from that in the clade including rodents and lagomorphs (mono- or dinucleotide P-SSR dominated; Figure 1). A similar observation was made based on a comparison of the dominant P-SSR category in intron and exon regions (Table 1). It is possible that the dominant category of P-SSRs in genomes or in the intron and exon regions of different clades has the potential to be used as markers for phylogenetic analysis at the order level in Euarchontoglires. However, the dominant P-SSR category should be used with caution for phylogenetic analysis given that most genomes of Scandentia species remain unavailable. As expected, trinucleotide motif repeats prevailed in CDS regions. Microsatellites are thought to be under selection in genomes, which is reflected in their distribution and abundance, both of which are much higher than expected by chance or random accumulation (Ellegren, 2004). Our results showed that (ACG)n, (CCG)n, and (AGG)n were the dominant trinucleotide repeat motifs across all Euarchontoglires genomes (Supplementary Table 5), which indicated that these preferred motifs might be transcribed repeatedly in the same amino acids and further affect the physical and chemical properties of the proteins (as reviewed by Saeed et al., 2016). Moreover, the prevalence of some specific repeat motifs in genomes has been shown to have specific effects on genome function (Deback et al., 2009; Behura and Severson, 2012; Bagshaw, 2017). Comparative analysis demonstrated that rodent genomes had more specific repeat motif categories in genic and intergenic regions than that of other clades. Therefore, one intriguing question that needs to be resolved in the further is the function of the high frequencies of these amino acids in specific species or clades of Euarchontoglires.

A previous comprehensive analysis constructed phylogenetic trees using SSR frequency and revealed that the distribution patterns of SSRs were evolutionarily conserved at the family level in insects (Ding et al., 2017). In this study, we are wondering whether the dominant repeat motif is also correlated with the phylogenetic relationships within Euarchontoglires. Surprisingly, we found that the dominant repeat motif type of P-SSR (mono- to hexanucleotide P-SSR) was highly conserved at all taxonomic levels in primates, but was more variable in rodents (Supplementary Table 5). However, the similarity in the dominant P-SSR motif types (e.g., hexanucleotide P-SSRs) among rodent species precludes their use as a molecular marker for phylogenetic studies within the genus in Rodentia (Figure 2). A possible mechanistic explanation for these taxon-specific signatures in primates and rodents is that SSRs could evolve differently among different lineages. There is a general consensus that the presence of SSRs in the genomes of organisms is biased toward certain specific repeat motifs in different clades (Alam et al., 2019; Manee et al., 2020; Qi et al., 2020). In this study, an enrichment trend of P-SSR density for certain motif types was observed among the five taxonomic orders (Figure 3), which was similar to the findings of a previous study (Srivastava et al., 2019). Primates are rich in (AT)n repeats, whereas in rodents, (A)n, (AC)n, or (AG)n repeats are the most common (Supplementary Table 5). It is noteworthy that the dominant motif types could be different within the same genus in rodents (e.g., *Microtus*). This is likely explained by the fact that genome nucleotide composition could shape the prevalence of certain repeat units (Tian et al., 2010). Furthermore, the variation in prevalent taxon-specific repeat units might exhibit different biological functions in different taxon clades. In human genomes, for example, dinucleotide microsatellites with repeat units consisting of 50% A or T show higher recombination rates than other types of dinucleotide microsatellites (Guo et al., 2009).

The GC content of mononucleotide P-SSRs was the lowest across all Euarchontoglires genomes studied in the present investigation, which was consistent with previous studies of bovids (Qi et al., 2018), beetles (Song et al., 2020), and forest musk deer (Qi et al., 2020), suggesting that similar selective constraints might operate upon the GC content of mononucleotides in different clades. However, the P-SSR category containing the highest GC content displayed no taxon-specific features in Euarchontoglires (Supplementary Table 10). Indeed, P-SSR categories containing the highest GC contents may even vary in phylogenetically related species, such as *Eulemur macaco* and *Eulemur fulvus*. Various relationships have been observed between the polymorphism levels of P-SSRs and its GC content in different species (Kelkar et al., 2008; Brandström and Ellegren, 2008; Payseur et al., 2011). For example, relationships between GC content and SSR polymorphism levels for di- and tetranucleotides are opposite in chickens (Brandström and Ellegren, 2008). Although the relationship between GC content and SSR polymorphism level in different taxonomic groups requires further investigation, the observed GC content variation suggests that the polymorphism of P-SSR varies widely and gives an indication of the genetic variation among Euarchontoglires species. Moreover, the average GC content of trinucleotide P-SSRs in genomes was 34.34%, while that in the CDS regions was 69.84% (Supplementary Table 9). Numerous studies have shown that the GC content of the DNA sequence is functionally important (Guo et al., 2009; Bhati et al., 2015; Kenigsberg et al., 2016); for example, some GC-rich SSRs may affect influence replication *via* their effects on DNA secondary structure (Nakagama et al., 2006; Bhati et al., 2015). Considering that trinucleotide P-SSRs are dominant in CDS regions, the GC content might be negatively associated with SSR variability to limit repeat number variation. The biological significance of the GC-rich bias of trinucleotides in CDS regions of Euarchontoglires requires further study.

SSRs with variable-length repeating motifs cause many human diseases (Yim et al., 2006; Wilkins et al., 2009; Sznajder et al., 2018). As expected, our scatter plot analyses revealed that the abundance of P-SSRs in different genomic regions decreased as the RCNs increased, and the RCNs of the microsatellites of corresponding motif lengths in the coding regions were lower than those in introns or in the whole genome (Supplementary Table 7). These results are consistent with the notion that the growth of long microsatellites is constrained by an upper length boundary that, when reached, sometimes results in large deletions (Vowles and Amos, 2006). The CV analysis of the RCN of P-SSRs demonstrated that patterns varied among different clade organisms. For example, a similar pattern was observed in bovids (Qi et al., 2018), but large variation was observed in beetles (Song et al., 2020). A possible explanation for this could be that the CVs of the RCNs of P-SSRs in species could be taxon-specific (Song et al., 2020), but the sample size could also considerably affect the results. Indeed, if all CV results derived from the 153 species are shown in one figure, no clear pattern of the CV could be observed (Supplementary Table 8). Moreover, a different pattern of CVs of P-SSRs was detected at every taxonomic level in Euarchontoglires (e.g., in *Eulemur* and *Microcebus*). Nonetheless, comparable results in genomes or in genomic regions of the five clades in this study could be observed when we plotted our results by using the average value of CV, e.g., the CVs of the RCNs of P-SSRs in CDS regions (Figure 4). Microsatellites are one of the most important sources of genetic variation, and the polymorphism levels are highly correlated with the repeat copy numbers of motif (Bagshaw, 2017). Therefore, interspecific differences in the CVs of P-SSRs can generate functional variability, and the variation in the CVs of RCNs could reflect fundamental differences among different organisms. Replication slippage (Saeed et al., 2016), heterozygosity (Amos, 2016) and varied environmental selection pressure are possible forces that could drive variation in CV trends among different species (Figure 4 and Supplementary Table 8).

### Functional Analysis of CDSs Harboring P-SSRs in Euarchontoglires Genomes

Recent studies have shown that the functional significance of SSRs in the modulation of gene expression and genome organization implies their functional conservation across species. An increasing number of SSRs have been developed from the transcriptomes of many organisms because of their importance as a source of functional markers (Park et al., 2019; Souza et al., 2020). However, the expression patterns of genes affected by many factors; thus, the SSRs derived from the transcriptome might be incomplete. In this study, we investigated the potential functions of CDSs containing P-SSRs in genomes of Euarchontoglires species by conducting GO and KEGG pathway enrichment analysis. It was intriguing that all the CDSs in different species containing P-SSRs were enriched in binding (GO:0005488) and transcription regulator activity function (GO:0140110), which is similar to the potential functions of the CDSs containing P-SSRs in beetles (Song et al., 2020). Moreover, KEGG enrichment analysis of genes containing SSRs indicated that transcription factors were the most well-represented pathways among Euarchontoglires species (Figure 6 and Supplementary Table 11). Transcription factors are key regulatory elements that affect gene expression which coordinate a lot of biological processes, such as development (Yan et al., 2018) and metabolism (Chisato et al., 2018). Therefore, it is reasonable to speculate that the genes containing P-SSRs might regulate the selective synthesis of certain proteins. In this study, our results demonstrated that the two most abundant transcription factor categories that containing P-SSRs in different clades of Euarchontoglires were identical (i.e., zinc finger protein and forkhead-box protein; Supplementary Table 12). Previous studies revealed that zinc finger transcription factors and forkhead-box transcription factors have key roles in various aspects of immune regulation (Coffer and Burgering, 2004; Sakaguchi et al., 2010; Zhang et al., 2019). Although some SSRs in genes could facilitate the binding of transcription factors (reviewed by Bagshaw, 2017), the potential roles of SSRs in transcription factors in Euarchontoglires species remain to be further explored. Moreover, SSRs can be regarded as mutational hot spots in genome sequences, and the resulting genetic variation has been reported to play a positive role in adaptive evolution (Kashi and King, 2006; Li et al., 2008; Gemayel et al., 2010). A similar analysis performed in the giant panda revealed that the genes possessing polymorphic coding SSRs were involved in digestion and metabolism, which may contribute to its special adaptive evolution to its specialized diet of bamboo (Cheng et al., 2019). Taken together, our work paves the way for further understanding and validating the function roles of the genes containing P-SSRs. Additional large-scale comparative functional analyses of CDSs containing P-SSRs in different organisms should be performed to assess the generality of the results documented in our study.

## Conclusion

Taxon-specific microsatellite distribution patterns were observed among different clades within Euarchontoglires. The dominant P-SSR categories in primates, tree shrews, and colugos were identical, but varied in rodents and lagomorphs. The enrichment of the most prevalent repeat motifs in specific clades was detected at the order level. However, the GC content and CV of P-SSRs varied greatly among different species at all taxonomic levels, suggesting that SSR variation might contribute to genetic variation among these mammals. We showed that the CDSs containing P-SSRs across all Euarchontoglires genomes were enriched for functions related to transcription. However, more work is needed to clarify the precise evolution and functional roles of taxon-specific P-SSRs in Euarchontoglires.

## Data Availability Statement

The original contributions presented in the study are included in the article/Supplementary Material, further inquiries can be directed to the corresponding author/s.

## Author Contributions

XS, CZ, and YW: conceptualization. XS and TY: writing-original draft. XS, TY, XZ, and YY: data curation. TY, XY, and JZ: formal analysis. All authors contributed to the article and approved the submitted version.

## Conflict of Interest

The authors declare that the research was conducted in the absence of any commercial or financial relationships that could be construed as a potential conflict of interest. The reviewer XZ declared a past co-authorship with one of the authors XS to the handling editor.

## Footnotes

1 http://www.ncbi.nlm.nih.gov/

2 http://www.genome.jp/kegg/kaas/

3 http://www.ehbio.com/Cloud_Platform/front/#/
